# Alternative Routes of Administration for Therapeutic Antibodies—State of the Art

**DOI:** 10.3390/antib11030056

**Published:** 2022-08-26

**Authors:** Aubin Pitiot, Nathalie Heuzé-Vourc’h, Thomas Sécher

**Affiliations:** 1 INSERM, Centre d’Etude des Pathologies Respiratoires, U1100, F-37032 Tours, France; 2 Faculté de Médecine, Université de Tours, F-37032 Tours, France

**Keywords:** therapeutic antibodies, administration routes, drug delivery, clinical trials

## Abstract

Background: For the past two decades, there has been a huge expansion in the development of therapeutic antibodies, with 6 to 10 novel entities approved each year. Around 70% of these Abs are delivered through IV injection, a mode of administration allowing rapid and systemic delivery of the drug. However, according to the evidence presented in the literature, beyond the reduction of invasiveness, a better efficacy can be achieved with local delivery. Consequently, efforts have been made toward the development of innovative methods of administration, and in the formulation and engineering of novel Abs to improve their therapeutic index. Objective: This review presents an overview of the routes of administration used to deliver Abs, different from the IV route, whether approved or in the clinical evaluation stage. We provide a description of the physical and biological fundamentals for each route of administration, highlighting their relevance with examples of clinically-relevant Abs, and discussing their strengths and limitations. Methods: We reviewed and analyzed the current literature, published as of the 1 April 2022 using MEDLINE and EMBASE databases, as well as the FDA and EMA websites. Ongoing trials were identified using clinicaltrials.gov. Publications and data were identified using a list of general keywords. Conclusions: Apart from the most commonly used IV route, topical delivery of Abs has shown clinical successes, improving drug bioavailability and efficacy while reducing side-effects. However, additional research is necessary to understand the consequences of biological barriers associated with local delivery for Ab partitioning, in order to optimize delivery methods and devices, and to adapt Ab formulation to local delivery. Novel modes of administration for Abs might in fine allow a better support to patients, especially in the context of chronic diseases, as well as a reduction of the treatment cost.

## 1. Introduction

Over the past 30 years, therapeutic antibodies (Abs) have been found to be valuable therapeutics [1]. A total of 6 to 12 new Abs are approved by the U.S. FDA and/or the EMA each year, and new molecules are reaching clinical trials every month [2]. Therapeutic antibodies are used in the treatment of numerous diseases, including infection, cancer, and autoimmune disorders, in which they have already demonstrated their efficacy [3,4].

The success of Abs is due to (I) a high level of specificity and affinity to their target antigen, (II) a favorable safety profile, and (III) a unique pharmacokinetic profile, supporting a longer half-life as compared to other drugs [5]. These characteristics have allowed Abs to move rapidly from pre-clinical studies to clinical trials, as observed during the COVID-19 pandemic [6].

From the historical full-length antibody, molecular engineering has enabled the development of multiple and diverse Ab formats, including multi-specific Abs, fragments, and conjugated Abs that are now extensively evaluated in clinical trials [7].

Due to their intrinsic biological properties, Abs have a specific interconnected pharmacokinetic and pharmacodynamic profile, which influence their absorption and biodistribution after administration [5]. Abs pharmacokinetics is linked to their route of administration [8]. Historically, Abs were delivered via intravenous (IV) injection. Nowadays, the subcutaneous (SC) route is often used for chronic diseases [9]. These systemic routes have the advantage of allowing the delivery of large amounts of Abs and to enable rapid systemic bioavailability. However, one of their drawbacks is the limited distribution from the site of injection via the blood flow to the diseased organ, which may result in limited Ab amount in the vicinity of the target antigen. Ultimately, this necessitates the injection of a high dose, which may be associated with potential toxicity and cost issues. Accumulating preclinical evidence has driven researchers to reconsider Abs’ route of administration in order to maximize their therapeutic index.

Alternative delivery methods, addressing Abs to the disease site (e.g., delivery of Abs in the lung to treat respiratory pathologies [10], or inside a tumor [11]) have emerged and progressed to the clinical trial stage. In theory, a higher concentration of the antibody at the target site should improve the therapeutic response, while lowering the concentration in neighboring healthy tissues, resulting in reduced side effects.

**Figure 1:**
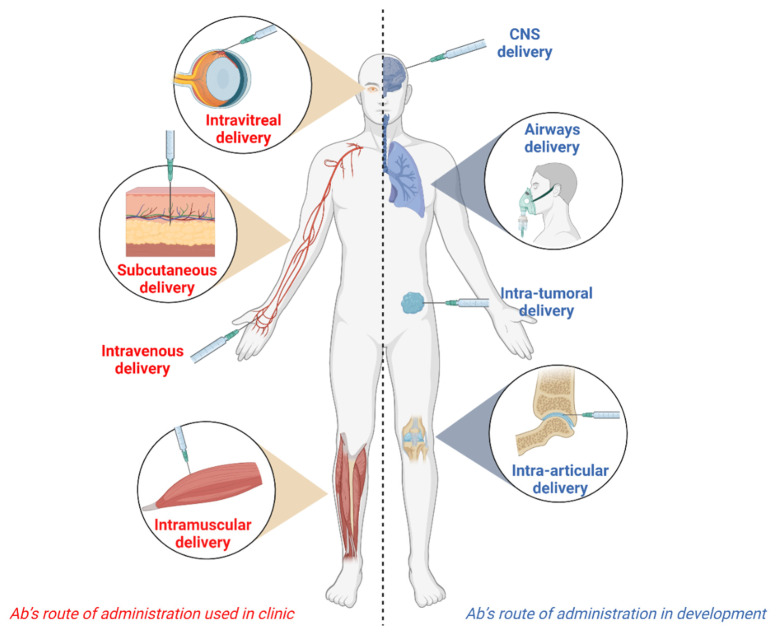
Routes of administration for therapeutic antibodies in Humans.

Here, we reviewed and analyzed the literature published as of the 1 April 2022, describing the different routes of administration used for the delivery of Abs. The IV route has not been considered in this review, being the subject of many reviews elsewhere [12,13] (Figure 1). Each section highlights the basics of the administration route, its application, the potential hurdles, and, when applicable, describes the Abs approved or under review by the regulatory agencies [14,15,16], and the molecules in the late stages of clinical trials. The publications were identified by searching MEDLINE and EMBASE databases. Ongoing clinical trials were found on https://www.clinicaltrials.gov/ (accessed on 1 April 2022) [17]. Our research strategy was based on the use of the keywords “Ab”, “mAbs” “therapeutic antibody”, “monoclonal antibody” “administration”, “delivery”, “injection”, “barriers” and “clinical trial”, as general criteria, and the keywords “subcutaneous”, “intramuscular”, “intravitreal”, “airways”, “inhalation”, “intra-tumoral”, “peri-tumoral”, “intra-articular”, “oral”, “intra-cerebral”, “intranasal”, “topical” for specific information on the route of administration.

## 2. Routes of Administration for Therapeutic Antibodies

### 2.1. The Subcutaneous Route: The Most Popular after IV Injection

#### 2.1.1. Fundamentals Related to the SC Route

After IV injection, the second most popular route for the delivery of antibodies is the subcutaneous (SC) route. It consists in the injection of Abs using a syringe and needle under the skin of patients at an angle of 90 °C, thus bypassing the barrier formed by the epidermis and dermis layers [18]. The choice of the anatomical site is important due to differences in dermal thickness which may reduce the absorption of the injected Abs. Nowadays, around 30% of the approved Abs are delivered by SC injection (Table 1).

If the delivery of drugs, mainly opioids, by SC administration, has been in practice since the middle of the 19th century, the administration of Abs by this route is recent. The first subcutaneous injected Ab was Adalimumab, used in the treatment of rheumatoid arthritis and approved by the FDA in 2002, and by the EMA in 2003 [19]. After this first success, and particularly since 2009, the number of marketed Abs delivered by the SC route has significantly increased. It is noteworthy that SC administration is already the standard route in the treatment of chronic diseases such as rheumatoid arthritis. Indeed, it allows self-administration and improves patients’ compliance. The SC route is mainly used for the delivery of Abs targeting interleukins such as TNF-α, critically involved in the development of rheumatoid arthritis (Adalimumab, Golimumab, Certolizumab pegol) or cytokine receptors such as the IL-17a receptor, involved in the progression of psoriasis (Brodalumab, Secukinumab, Ixekizumab) [20].

The development of Abs intended for a subcutaneous injection necessitates understanding the physiology of the skin. After injection, the drug reaches the hypodermis interstitial space between the dermis and the deep fascia covering the muscle tissue. This layer is composed of adipose tissue, blood, lymph vessels, and resident immune cells such as fibroblasts and macrophages. All components are enmeshed in an extracellular matrix (ECM) network, rich in collagen, elastin, and glycosaminoglycans [21]. To pass into the systemic compartment (via either the blood capillaries or lymphatic vessels), and thus reach their target, Abs have to diffuse through the ECM, which constitutes both a physical and chemical barrier. The fate of the Ab is dictated by its size, charge, and affinity with transporters. Despite the presence of the positively charged collagen fibrils, the hypodermis interstitial space displayed an overall negative charge due to important concentrations of hyaluronic acid and chondroitin sulfate, two major glycosaminoglycans of the hypodermis ECM, which are negatively charged. The global negative charge of ECM favors the transport of negatively charged drugs thanks to electrostatic repulsion [22]. However, the majority of therapeutic Abs are positively charged. Once the ECM is traversed, drugs may enter the systemic circulation by two different mechanisms. Molecules smaller than 16 kDa diffuse directly into the bloodstream, taking advantage of the permeability of the vascular endothelium [22]. However, Abs, along with drugs with a higher molecular weight, are absorbed by convection into lymphatic vessels. Thus, the subcutaneous route is particularly interesting to target lymphoid cells and the molecules they secrete. Abs in the lymphatic vessels pass to larger lymphatics and then reach the blood vascular system, from where they diffuse throughout the body.

If the development of Abs for subcutaneous injection is quite challenging, multiple factors explain the attractiveness of this route as compared to other parenteral ones. In the hypodermis, the walls in the fat lobule are thinner than those in the dermis, which facilitates the diffusion of drugs into blood capillaries [23]. Moreover, the absence of antigen-presenting cells in the hypodermis, usually present in the top layers of the skin (Langerhans cells and/or macrophages), may decrease the immunogenicity of the antibody. Thus, an increasing number of Abs delivered by SC are being developed, allowing a quicker delivery time of administration as compared to IV injection, enabling longer dosing intervals and, in fine, reducing the frequency of administration. In addition, SC administration is less invasive and painful than IV injection [22] and allows self-delivery at home [24]. Thus, the subcutaneous route may improve patient comfort and compliance, which is critical for the treatment of chronic diseases, and may be associated with a reduction in treatment costs, consuming fewer healthcare resources.

#### 2.1.2. Abs Approved for Subcutaneous Delivery

Abs approved for subcutaneous administration must be formulated at a high concentration, thus necessitating a careful control of their stability and formulation viscosity. Different strategies have been considered to ensure the efficient absorption and bioavailability of Abs after hypodermis injection. They include, but are not limited to, the increase in delivered Abs concentration (e.g., SC administration limiting the injection volume to 1–2 mL [25]), the development of specific formulations to reduce physical and chemical destabilization (e.g., the use of polysorbate preventing aggregation and particle formation [26]) and the development of novel administration devices (e.g., the autoinjectors enabling a faster delivery for larger concentrations of Abs [27]). Those strategies have led to the approval of around 40 different Abs (Table 1).

A major concern for SC injection is the isoelectric point (pI) of Abs, found between 7 and 9, making Abs positively charged at the physiological pH. A study by Bumbaca Yadav et al., showed that positively charged Abs present a reduced bioavailability by 31%, while their negatively charged counterparts demonstrate enhanced bioavailability up to 70% after SC administration [30]. Another study found that the reduced bioavailability of Abs delivered subcutaneously is due to their interaction with ECM components, thus limiting the amount of Ab reaching the vascular compartment [31]. Moreover, the overall negative charge of the hypodermis interstitial space increases the interaction of ECM components with water molecules resulting in a low hydraulic conductivity and limiting the subcutaneous injection volume [32]. To circumvent this serious issue, one strategy consists in combining Abs with hyaluronidase. Hyaluronidase degrades hyaluronic acid, lowering the amount of negatively charged molecules and enhancing the bioavailability of Ab after SC injection [33]. Moreover, combining Abs with hyaluronidase may facilitate bulk fluid flow and improve the pharmacokinetic profile after SC injection [34], as demonstrated in multiple clinical studies [35,36,37]. Based on these results, the regulatory agencies approved Rituximab, Trastuzumab, and Daratumumab in combination with recombinant human hyaluronidase (rHuPH20), in 2017 (Rituxan Hycela/mAbThera s.c), 2019 (Herceptin Hylecta), and 2020 (Darzalex Faspro), respectively.

These encouraging results have fueled the repurposing of Abs approved for delivery by IV injection to this novel modality of administration. Notably, Tocilizumab ((Ro)-Actemra), an antibody used in the treatment of rheumatoid polyarthritis, was formulated for the SC route, in response to patient demand, and to allow a less invasive route for a treatment usually delivered monthly [38]. It is noteworthy that multiple studies have demonstrated the absence of significant differences between the IV and SC routes of administration for Abs, thus making SC a legitimate option for patients [37,38].

#### 2.1.3. Abs in Clinical Development for the SC Route

The clinical development of subcutaneously-delivered Ab concerns either de novo development, expansion of the disease target, and/or new formulation for already approved Abs. A high number of those Abs are currently found in clinical trials. Here, we listed subcutaneously delivered Abs either in active phase 3 trials or under review by regulatory agencies (Table 2).

Novel developments include Fasinumab, a recombinant fully human IgG4, targeting the nerve growth factor (NGF) and evaluated for pain relief in patients suffering from osteoarthritis (OA). A phase 2b/3 trial showed that Fasinumab provides improvement in OA pain and motor function, even in patients that are non-responsive to analgesics [39]. The drug approval is pending an evaluation by the FDA (NCT03161093; NCT02683239). In the meantime, studies are also investigating lower doses of Fasinumab in patients with knee or hip OA. 

The repurposing of IV delivery approved Abs for SC application in a disease context different than the original approval is also investigated. For example, Ofatumumab (Arzerra^®^, Novartis) is a monoclonal antibody targeting CD20 and causing cytotoxicity in cells expressing CD20. It was first approved in 2010 for the treatment of certain chronic lymphocytic leukaemia by IV injection, and has been reformulated (Kesimpta) for SC administration and evaluated in patients with relapsed multiple sclerosis. Two ongoing phase 3 trials, OLIKOS (NCT04486716) and ARTIOS (NCT04353492) are evaluating the efficacy, safety, and tolerability of the SC drug in patients with relapsing multiple sclerosis, all of whom are transitioning from a CD20 Ab therapy (Rituximab or Ocrelizumab), or dimethyl fumarate therapy [40].

Many Abs have been developed or repurposed as emergency treatments since the beginning of the SARS-CoV2 pandemic, and target either the virus or the host inflammatory response. Among them, REGEN-COV2 comprising Casirivimab and Imdevimab has been approved for emergency use by IV infusion and is now undergoing regulatory review, for its use by SC administration to treat and prevent SARS-CoV-2 infection in non-hospitalized individuals [41]. The first phases of its clinical investigation showed a significant efficacy with improved survival. A phase 3 trial is also in progress to evaluate its potency for the prevention of COVID-19 in immunocompromised patients (NCT05074433).

#### 2.1.4. Conclusion and Perspectives Regarding the Subcutaneous Route

The relevance of the SC route for the administration of Abs has been illustrated by several clinical successes. However, the bioavailability of Abs delivered subcutaneously remains difficult to predict. Advances in preclinical models would be necessary to investigate the fate of Abs at the SC injection site and their diffusion into the blood/lymphatic compartment. Interestingly, novel in vitro tools have been developed to predict the in vivo absorption of biopharmaceuticals after SC injection, by modeling the environmental changes an Ab will experience after its injection. Among those, Scissor (Subcutaneous Injection Site Simulator) device provides a tractable method to study the fate and the pharmacokinetics of biopharmaceuticals once in the hypodermis [42,43]. Nevertheless, as no in vitro model is yet accurate enough, the pharmacokinetics of Abs still relies on in vivo studies.

Formulating Abs for the SC route remains challenging, as formulations need to afford high concentration with low viscosity, aggregation and immunogenicity [22]. Biotechnological platforms have been developed to support the switch from intravenous infusion to SC delivery, using proprietary excipients and proteins, allowing the reduction of ionic strength and hydrophobicity areas of the molecule, thus limiting aggregation when the Ab is highly concentrated.

### 2.2. The Intramuscular Route: The Favorite Choice for Infectious Diseases? 

#### 2.2.1. Basics of the Intramuscular Route

If the intramuscular route (IM) is often used for the administration of vaccines, this modality is rarely considered for the delivery of Ab. The IM injection consists in administering a drug deep into the muscle mass, where the blood supply allows a rapid and better absorption than the subcutaneous route. As for the SC route, the drug is administered via a syringe and needle into the skin at an angle of 90°. However, where the SC route uses small length- and diameter-needles, IM injection requires longer needles—2.5 to 4 cm—to bypass the different layers of the skin. The volume of administration is relatively low ~2–5 mL, depending on the muscle chosen for the injection, necessitating a more concentrated product than for IV administration. The preferred injection sites are the deltoid muscle in the upper arm, the *vastus lateralis* found at the front of the thigh toward the outside of the leg, especially for IM injection in young children [44], the ventrogluteal muscle of the hip, the safest site for adults and children older than 7 months, and finally the dorsogluteal muscles of the buttocks, albeit less used nowadays due to potential injury to the sciatic nerve.

The pharmacokinetics of a drug injected by IM follows a specific sequence: dissolution rate, solvent supply, phase transfer, and diffusion to the vascular system. Therefore, injection depth is an important parameter which influences the absorption rate of the drug after IM injection. Consequently, a too superficial injection will deliver the drug either SC, or into the fat layer, retarding the action of the drug. The absorption will also depend on the muscle mass and its vascularization. Patients with muscular atrophy see a delay in drug absorption, as well as an increase in the risk of neurovascular complications. These anatomical parameters also influence the rapidity of action of the drug [44].

#### 2.2.2. Ab Approved for Delivery by the IM Route

One of the most threatening pathogens responsible for lower respiratory tract infection in children is the respiratory syncytial virus (RSV). Nearly every infant develops an RSV infection during their childhood. A total of 60% of them have an immune system mature enough to control the infection [45]. However, between 15 and 40% of infants, especially preterm infants, develop a more serious airway infection, which may eventually lead to bronchiolitis and pneumonia. Although no vaccine or specific RSV treatment exist to treat the infection (apart from general anti-viral therapy), a passive immunization may limit the infection in young infants with high risk factors. In 1998, the FDA approved Palivizumab (Synagis) [46], a humanized monoclonal IgG1 antibody targeting the glycoprotein F on the RSV virus, which is responsible for membrane fusion and infection of the host cell. Intramuscular injection of palivizumab is recommended for premature infants and young children with heart or lung comorbidities, and injections usually start before the expected RSV epidemic season [47,48]. Intramuscular delivery was chosen to facilitate delivery and limit invasive risks to the young recipients of the treatment. Aggregated clinical data have established the half-life of Palivizumab administered via intramuscular injection to be around 17–27 days, with a mean of 20 days, in infants younger than 24 months. As such, a monthly administration during the epidemic season is necessary to protect effectively against RSV. Palivizumab prophylaxis has shown a significant decrease in the hospitalization, ICU stay, and mortality [49,50]. Unfortunately, its high cost limits the number of children who benefit from this prophylaxis protection, especially in low-income countries [51]. Although Palivizumab is not licensed for the treatment of RSV disease, it has paved the way to the clinical use of intramuscular injection, and has shown some potential to facilitate everyday care, promoting a less invasive route for patients, in particular infants, in whom IV injection is more complicated [52]. 

#### 2.2.3. Abs in Clinical Development Delivered by the IM Route

The IM injection allows a more rapid absorption and onset of action compared to SC delivery: mathematical in silico models associate the IM route with higher drug concentration on target and shorter time to reach the peak of concentration as compared to SC delivery. This route benefits from the long experience of vaccine administration. As shown below, several anti-infective Abs delivered by IM administration are close to reaching patients (Table 3).

In 2021, Sotrovimab (Vir Biotechnology—GlaxoSmithKline) obtained an emergency use authorization (EUA) from the FDA for the treatment of mild-to-moderate COVID-19. However, since May 2022, and due to its inefficacy against the BA.2 variant of SARS-CoV-2, its authorization has been revoked [53]. A phase 3 trial (COMET-TAIL, NCT04913675) which has established that the intramuscular injection of Sotrovimab had a similar clinical effectiveness as compared to an IV injection is still active. 

SYN023, a therapeutic cocktail comprising two monoclonal Abs, CTB011 and CTB012, is under development for post-exposure prophylaxis to rabies. Nowadays, the standard of care treatment after rabies infection is an on-site (wound injection) and IM administration of anti-rabies immunoglobulin (RIG), called post-exposure prophylaxis, followed by four doses of the vaccine, delivered by IM or intradermal routes. The full dose of RIG facilitates rapid protection until the immune system can produce its own immunoglobulins thanks to the vaccination. However, the current RIG products are limited and expensive, thus their use is limited in high-burden countries [54]. SYN023, administered by IM injection, targets protein residues found on human rabies virus, and displays a neutralization capacity equivalent or superior to RIGs, even at a lower dose [55]. The ongoing phase 3 trial will provide additional information regarding the efficacy of SYN023 in populations at risk of rabies infection.

Ibalizumab-uiyk (Trograzo) is an anti-HIV monoclonal antibody, approved in 2018, for the treatment of patients with resistant forms of AIDS by IV injection. It targets CD4 protein expressed by T cells, inhibiting the entry of HIV. As a “first class medication” (defined by the FDA), the use of Ibalizumab-uiyk needs to be expanded across the world and for patients reluctant to receive IV injection or where the access to medical facilities and personnel qualified to administer IV injections is limited. A phase 3 is currently evaluating the IM route in comparison to IV injection, notably in terms of safety, pharmacokinetics, and limiting the spread of infection (NCT03913195). 

#### 2.2.4. Conclusions and Perspectives on the Intramuscular Route

The IM injection is usually considered as a rescue route of administration when other ways to deliver drugs are not appropriate. For example, drugs inducing vein irritation or sensitive to oral digestion have been moved from IV and *per os* injection, respectively, to IM injection. Among other advantages, IM administration is associated with a quicker and more uniform absorption of the drug as compared to other routes, and it is considered to be as efficient and potent as IV injection, with less invasive characteristics. Even with these potential advantages over other routes of administration, the IM route is still underestimated for Abs. The development of depot injections may represent an opportunity for the expansion of the IM route. Depot injections are a slow-release form of medication usually implanted in the muscle. They are already used for neuroleptic drugs (fluphenazine), prolonging their pharmacological effect. Moreover, they allow a sustained delivery over time [56] which may be of particular interest regarding the treatment of chronic diseases.

### 2.3. The Intraocular Route: An Invasive Route for Ophthalmic Disorders

#### 2.3.1. Principles Related to the Intraocular Delivery of Abs

With the population aging, the prevalence of ophthalmic disorders has increased over the last 20 years and is estimated to reach several hundred million in the next decades [57]. The most prevalent age-related eye disease is macular degeneration (AMD), accounting for 196 million patients, in 2020. This continuously evolving disease affecting the retina leads to near blindness, due to the overexpression of vascular endothelial growth factor (VEGF), and has no curative treatment. During AMD, VEGF expression is abnormally increased promoting the development of microvessels, leading to micro-hemorrhage and the progression of the disease.

Drug delivery to the retina is challenging, and various routes of administration have been considered to circumvent the complexity of the eye anatomy and improve the therapeutic index of drugs. The eye comprises an anterior and a posterior part. The anterior part, closer to the external environment, composed by the cornea, the conjunctiva, the ciliary body, the aqueous humor, lens, and the lachrymal system, forms a static barrier which prevents the access of foreign particles to the eye. Topical administration on this side results in low bioavailability of the drug (less than 3%) inside the eye, and an even lower in the posterior part [58,59]. Penetration enhancers may be required to improve topical administration [60]. The posterior part encompasses the vitreous humor, retina, sclera, and choroid. The main barrier on this side is due to the cornea, a negatively charged tissue repulsing negatively charged drugs [61]. The intra-ocular space is also protected by two additional obstacles, the blood-aqueous, and the blood-retinal barriers [62], both composed of tight junctions which limit the transfer of drugs from the blood compartment to the eye, especially considering high molecular weight biotherapeutics. In this context, the local delivery of Ab, directly in the internal parts of the eye by intravitreal injection has been developed to provide a high amount of drug in the retina and the vitreous compartments. 

The intravitreal administration is an invasive procedure consisting in the injection of the Ab, posterior to the limbus, thanks to an incision through the sclera at a specific angle of 30°. The only approved syringe systems for intravitreal injection are the ones already prefilled with Ab. Once the needle is in position, the Ab solution (with a limited volume, ~100–200 µL) is slowly applied into the vitreous cavity, avoiding damage to the retinal surface [63].

#### 2.3.2. Approved Abs by Intravitreal Administration

Up to now, three Abs delivered by intravitreal injection have been approved for the treatment of AMD and equivalent ophthalmic diseases (Table 4).

Ranibizumab (Lucentis), a humanized Fab fragment, [64] and Brolucizumab (Beovu), a humanized single-chain antibody fragment, both targeting all isoforms of VEGF-A [65] have been approved in 2006/2007 and 2019/2020, respectively. Recently, Faricimab (Vabysmo), a bispecific antibody targeting both VEGF-A and ANG-2 [66], has been approved by the FDA, for the treatment of AMD and diabetic macular edema. All these Abs aim at blocking the neo-vascularization, which is the main pathological process associated to retinal diseases [67]. It is noteworthy that Bevacizumab (Avastin), the first anti-VEGF-A antibody approved for cancer treatment, was also considered as an off-label indication for AMD by intravitreal injection, due to its more affordable price [68]. Unfortunately, it did not attain the required professional or political consensus to obtain final approval [69].

The pharmacokinetics of these Abs has been extensively studied. In aqueous humor, Bevacizumab and Ranibizumab were shown to have a half-life of around 10 days, and 7 days, respectively [70,71]. Abs concentration peaked on the first day of injection, and then rapidly declined. The short intravitreal half-lives implied frequent injections to ensure optimal effect, increasing the potential risks of side-effects. Interestingly, Faricimab demonstrated longer duration of action in a phase 3 clinical trial, with a sustained efficacy over 16 weeks post-injection [72]. 

To better understand the PK-PD of Abs after intravitreal administration, Mazer et al. developed a mechanistic model of intravitreal pharmacodynamics of anti-VEGF in the eye and demonstrated the interrelationship between the half-life of the Ranibizumab and the in vivo VEGF kD [73]. They also showed that the ocular Ab t1/2 was proportional to the hydrodynamic radius of the antibody, and the radius of the vitreous globe. Thus, the biodistribution and absorption of Ab depends on the physiological state of the eye, with a potential decrease in Abs efficacy for older eyes, or eyes impaired with either globe abnormalities or underlying pathologies (e.g., myopia, or hypermetropia) [74].

#### 2.3.3. Abs in Clinical Development for Intravitreal/Intraocular Delivery

The global aging of the human population accounting for an increased prevalence of AMD, or retinal vein occlusion along with the effectiveness of intravitreal administration, has paved the way for further developments of intravitreally/intraocularly-delivered Abs products (Table 5). 

It is noteworthy that several ongoing trials are further evaluating currently approved Abs delivered by the intravitreal route (Table 4) to either identify their long-term effects in patients with AMD (NCT04777201) or evaluate their efficacy in other ophthalmic diseases (NCT04740905). Notably, Faricimab, after being recently approved for AMD is currently being investigated in macular edema and branch retinal occlusion. 

KSI-301 is a humanized antibody against VEGF-A conjugated to a biopolymer, under investigation for the treatment of different ophthalmic disorders [75,76]. Apart from its excellent safety and better efficacy than the current treatment using the fusion protein Aflibercept, the phase 1 trial showed longer durability, allowing patients to achieve treatment-free intervals of 4 to 6 months.

Few novel drugs delivered by intravitreal injection are currently being developed. However, biosimilars are currently under investigation. HLX04-O, a biosimilar of Bevacizumab, is a monoclonal antibody under development by Shanghai Henlius Biotech, targeting VEGF to treat AMD. Contrary to the original molecule, this biosimilar has been developed *de novo* for intraocular administration. Early-stage clinical trials, analyzing the safety and toxicity of the molecule delivered intravitreally, showed promising results, similar to the results obtained with Bevacizumab. As of April 2022, a Phase 3 trial was launched with the administration of HLX04-O to a first patient [77]. MW02, a recombinant anti-VEGF humanized monoclonal antibody, is under phase 2 and 3 evaluation. The trials compare its efficacy and safety to Ranibizumab (Lucentis) (NCT05297292).

#### 2.3.4. Conclusion and Perspectives on the Intravitreal Route

While anatomic barriers prevent Abs access to the eye from the blood compartment, intravitreal injection has demonstrated efficacy and has become the standard-of-care in the treatment of eye disease. Although it is invasive, it is associated with limited side-effects such as uveitis and vitreitis [78], and a higher therapeutic index. To support the development of intravitreally-delivered Abs, advances are necessary in formulation and medical devices, to allow prolonged action, longer periods between two injections, and better tolerability.

For example, the Port Delivery System (PDS) has been proposed for the delivery of Ranibizumab. It consists of a device surgically implanted into the vitreous cavity, allowing continuous delivery of the Ab. It dispenses the need for frequent intravitreal injections, thereby reducing invasiveness for patients. Its clinical evaluation is on-going and the first results for patients suffering from neovascular age-related macular degeneration are promising, showing equivalent control of the disease as compared to the standard care treatment, a monthly intravitreal injection of Ranibizumab (NCT04657289). 

The repertoire of excipients recognized as safe by the intravitreal route is limited due to ocular toxicity. For instance, only polysorbate (80 and 20) and sugar residues have been included in the formulation of Brolucizumab, Faricimab, and Ranibizumab [79,80]. Similar to what is observed in SC administration, Abs diffuse poorly into the vitreous cavity due to their positive charge [81]. Multiple parameters may influence the diffusion of Abs inside the eye, including: (i) the age of the eye, which can be associated with a change in the viscosity of the vitreous humor; and (ii) the quality of the lens. Patients with cataract surgery might experience a more rapid clearance of Abs from the eye, as observed with intravitreal delivery of antibiotics [82].

Overall, intravitreal injection is quite challenging, and inter-individual variability will dictate the fate and the activity of the antibody inside the eye. Like other local routes of administration, intravitreal injection of Ab provides effective on-target treatment, while limiting systemic deleterious exposure, and thereby improving Ab therapeutic index.

### 2.4. Inhalation: An Alternative Route for Respiratory Diseases

#### 2.4.1. Rationale of Delivering Abs through the Airways

Respiratory diseases are a major worldwide public health issue. They represent the fourth most common cause of death worldwide, which is mainly attributable to lung cancer, lung inflammatory diseases (e.g., chronic obstructive pulmonary disease (COPD)), and lower respiratory tract infections. The increase in antimicrobial resistance, seasonal virus outbreaks, newly emerging pathogens, or atmospheric pollution have severely complicated the management of airway diseases. Owing the success of Obiltoxaximab/Raxibacumab against pulmonary anthrax and Benralizumab against asthma, Abs have emerged as powerful therapeutics to tackle multiple respiratory diseases [83]. However, Abs administration through systemic routes led to a low bioavailability in the airway compartment [84,85]. This may limit the efficacy of Abs to treat respiratory diseases, if their target antigen primarily acts within the respiratory organ, while exposing the rest of the body to potential side effects [86].

Oral inhalation is the gold standard route of administration for small molecules commonly used for the treatment of inflammatory diseases (asthma, COPD). It allows direct drug access—as an aerosol—to both the upper and lower respiratory tract. It is associated with a rapid onset of action and a better therapeutic index as compared to parenteral injections [87,88]. Abs aerosolization is generally difficult since they are highly sensitive to mechanical, thermal, and physical stresses occurring during aerosol generation. Two types of devices have been evaluated for the oral inhalation of proteins: dry-powder inhalers, which deliver solid aerosol, and nebulizers, delivering liquid-formed aerosols. So far, metered-dose inhalers are not recommended for proteins due to the use of propellants [89].

#### 2.4.2. Challenges Associated with the Clinical Development of Inhaled Abs 

Researchers and pharmaceutical companies have been interested by local delivery of Abs through the airways for the last 20 years [90]. However, the first attempts were unsuccessful [91] and may be explained by multiple factors:

− Abs instability during aerosolization/spraying, resulting in aggregation and thereby potential alteration of activity and immunogenicity concerns [86,92,93].
− Relevant target antigen, which must operate within the respiratory tract and be critical in the pathophysiology of the respiratory disease
− Selection of the appropriate population, which may benefit from inhaled Abs.
− Biological barriers, which may impair Abs PK and activity [94].

Several inhaled Abs are in early clinical trials, after preclinical studies showing promising efficacy in animal models (for example anti-IL-13 Fab for the treatment of asthma [95]). A few inhaled Abs are in phase 2 and 3 clinical evaluation, notably for the treatment of SARS-CoV-2 infection (Table 6).

CSJ117 (Ecleralimab) is an inhaled TSLP inhibitory antibody fragment developed to treat moderate to severe asthma [96] and COPD. The antibody is provided as a powder in hard capsules to be delivered daily in the lungs using a dry powder inhaler. First results showed promising results, with CSJ117 having the ability to attenuate airways inflammation in asthma patients.

The recent SARS-CoV-2 pandemic has brought attention to the inhalation route, and several inhaled anti-SARS-CoV-2 Ab are being tested in the clinic by oral inhalation. Among the most advanced is a combination therapy developed to tackle the emerging SARS-CoV2 mutants, showing promising results. CT-P63 and CT-P59 (Regdanvimab) are monoclonal Abs, both targeting the receptor binding domain of the spike protein of SARS-CoV-2. More than just eliciting a neutralizing response, these Abs have the property to trap viral particles within the mucus, promoting their elimination by mucociliary clearance [97].

It is noteworthy that one molecule is currently under development by nasal inhalation, to address the Ab into the nasal cavity rather than in the lungs (COVI-DROPS).

#### 2.4.3. Conclusion and Perspectives on the Inhalation Route

Although airway delivery of Abs has been demonstrated as feasible and promising in preclinical studies, the benefits of Abs inhalation have not materialized yet in the clinical setting. A major advantage of the inhalation route is on the possibility of self-administration of Abs treatment for non-hospitalized patients, thereby reducing healthcare costs, and increasing patient comfort [98]. However, groundwork is still necessary to optimize Abs design and formulation, inhalation devices, as well as improving our knowledge of the physical and biological barriers that may impair the therapeutic response to inhaled Abs. 

### 2.5. Intra-Tumoral Administration: Overcoming the Tumor Stromal Barrier for Anti-Cancerous Abs

#### 2.5.1. Overview of the Barriers Associated with Tumor 

Abs used for the treatment of cancer are systematically delivered through IV injection. Their targets cover a wide range of proteins. They will promote the blocking of signals needed for cancer cell survival/growth, induce cancer cell destruction when coupled with toxins, or mark the tumor so that the immune system will better recognize and destroy it. Recently, the development of Abs targeting immune checkpoints has changed the treatment paradigm in many cancers. Abs targeting immune checkpoints inhibitors (ICI) block the immune “off” signal induced by the tumor cell in order to prevent the immune system from destroying the cancer, thus allowing a natural anti-cancerous immune response, and a higher survival for patients [99]. Systemic anti-cytotoxic T-lymphocyte-associated protein 4 (CTLA4) Ab was the first licensed ICI against advanced metastatic melanoma [100], and was then quickly followed by anti-programmed cell death ligand and protein 1 (PD-L1/PD-1) Abs. However, if ICI antibodies have shown efficacy in patients with melanoma or non-small cell lung cancers, many cancers remain refractory to them. In addition, if the systemic ICI treatment has undeniable advantages, such as predictable pharmacokinetics, it is associated with hazardous on-target/off-tumor side effects, including widespread inflammation. Thus, improving the tumor targeting of ICI Abs appears to be an evident goal. 

In fact, most solid tumors are poorly reachable from the systemic circulation, due to multiple surrounding barriers [94]:

− Densified tumor-associated ECM with the overexpression of collagens, and fibronectin, preventing the diffusion of Abs [101].
− Abnormal growth of blood vessels in the vicinity of the tumor resulting in non-vascularized, inaccessible areas for Abs coming from the blood circulation [102].
− Disorganized vessel structure associated with blood flow resistance, impeding the transport of the drug [103].

Consequently, several strategies have been proposed to limit the side effects of Abs targeting ICI, as well as optimizing their bioavailability within the tumor environment. A novel accurate method of injection consists in the delivery of Abs intratumorally (IT), allowing access to the tumor vicinity, circumventing extracellular barriers, and in theory improving on-target efficacy. Intra-tumoral injection is performed in a clinical environment by a physician, under the guidance of imagery (e.g., ultrasound, computed tomography (CT), or endoscopy) [104].

#### 2.5.2. Abs in Clinical Development by Intra-Tumoral Injection

Preclinical studies have established that local administration of anti-CTLA-4 Abs was able to restore an anti-tumor response, eradicating both local and distant tumors, and used a significant lower amount of Abs as compared to IV Ab [105,106]. Thanks to the success of those pre-clinical studies, novel intratumorally-delivered Abs are under clinical evaluation (Table 7).

In 2019, an anti-CD40 agonist antibody (ADC-1013) was developed and clinically evaluated in solid tumors after intra-tumoral administration (NCT02379741). The results from the phase 1 study showed a good safety profile and were associated with positive pharmacodynamic responses [107].

Ipilimumab is a human anti-CTLA4 antibody already approved for the treatment of metastatic melanoma and given by IV injection. A phase 1 study showed that Ipilimumab in combination with IL-2, given intratumorally, was well tolerated and was able to generate anti-tumoral responses in the majority of patients [108]. A phase 2 study is currently evaluating the tolerance of Ipilimumab IT and Nivolumab IV for the treatment of stage III/IV melanoma patients (NCT02857569). Moreover, a phase 2/3 trial is comparing Pembrolizumab and/or Ipilimumab in patients with advanced solid tumors after intra-tumoral injection and IV infusion. (NCT03755739).

#### 2.5.3. Conclusion and Perspectives on the Intra-Tumoral Route

Intra-tumoral administration of anti-cancerous Abs has multiple advantages, especially in reducing the harmful side effects associated with on-target/off-tumor of Abs after IV injection. In addition, local administration, within the tumor environment, may promote a superior priming of T-cells already on-site, and a multi-clonal response [106].

For now, clinical validation of intra-tumoral delivery of Abs is still in the preliminary stages. Further work is needed to optimize the dosage and the formulation according to the type and the stage of the tumor lesion. In addition, this route of administration is operator-dependent, which means that a subtle change in needle position may change the outcome of the administration. The development of new live imaging tools may improve injection reproducibility. Combinatorial approaches with intra-tumoral and intravenous treatment, or tumor ablation, are also being investigated and may represent a promising therapeutic alternative [109].

### 2.6. Intra-Articular Administration: A New Hope for the Relief of Joint Pain

#### 2.6.1. Overview of Joint Physiology 

Joints degeneration is one of the leading causes of permanent motor disability worldwide requiring long-term therapeutic treatment. Among them, osteoarthritis (caused by trauma to the joint cartilage), rheumatoid arthritis (autoimmune disease where immune cells attack the joints), and gout (joint inflammation due to excess of uric acid), are the most common forms [110]. Standard care includes systemic administration of analgesic and anti-inflammatory agents, including anti-TNF-α (Infliximab, Etanercept, Adalimumab) molecules and anti-IL1β Ab (Canakinumab) [111]. However, although symptoms show substantial improvements thanks to these treatments, non-responsive patients and side effects are increasing with time [112]. 

Efforts have been made in recent years toward the development of intra-articular (IA)-delivered treatments, with multiple benefits such as a better bioavailability and a reduced systemic exposure [113]. Intra-articular injection is performed using syringe/needle into a joint. To help the guidance of the needle, ultrasound or fluoroscopy techniques can be used. The development of drugs dedicated to IA injection necessitates understanding the anatomy and physiology of the joints, to identify the key parameters influencing the pharmacokinetics and pharmacodynamics of the drug.

Synovial joints, or diarthrosis, joins bone endings and hyaline cartilage with a fibrous capsule delimitating the synovial cavity filled with synovial fluid. Synovial joints are particularly affected during the degeneration processes [114]. Cartilage is an avascular tissue composed of chondrocytes embedded in a negatively charged ECM which make this tissue impermeable to molecules bigger than 50 kDa (such as antibodies) depending upon their charge and conformation. Consequently, the cartilage is inefficiently targeted by drugs administered systemically, which first need to reach the synovial fluid, before diffusing through cartilaginous ECM. To enter the joints, the drug needs to pass through the capillary endothelium of the synovium, the ECM of the synovial intima, and the synoviocytes composing the synovial membrane. Both cellular layers are highly fenestrated, allowing high diffusion of molecules smaller than 10 kDA. For larger molecules, the fenestration will allow a size-dependent diffusion, slowing the passage of large molecules such as antibodies [115]. During inflammation, there is an increased permeability of both capillary and synovial membranes, allowing macromolecules to reach the synovial compartment. However, joint inflammation will also accelerate synovial clearance [116]; studies have revealed a mean clinical half-life of around 3 h for an anti-inflammatory antibody, not leaving enough time for an optimal action of the antibody.

#### 2.6.2. Abs in Clinical Development for IA Administration

Despite some promising features, intra-articular delivery of therapeutic antibodies remains rare. Studies performed 20 years ago have shown conflicting results regarding IA delivery of Infliximab depending on the disease, with positive outcomes in patients suffering from ankylosing spondylitis [117], while there was no positive effect in patients with acute joint inflammation [118]. In 2007, the clinical evaluation of intra-articularly-delivered Infliximab in patients suffering from intractable knee monoarthritis, reached a phase 3 clinical trial but the development was stopped in 2015 due to the insufficient recruitment of patients [117] (NCT00521963). More recently, two novel intra-articularly-delivered Abs have entered clinical evaluation (Table 8).

AMB-05X is a fully human antibody targeting colony-stimulating factor I (c-FMS), a protein overexpressed in many cancers, and on tumor-associated macrophages. This antibody entered a clinical trial phase 2 (NCT04731675), to treat patients suffering from tenosynovial giant cell tumor, and pigmented villonodular synovitis, two afflictions of the knee, after IA delivery. The ongoing study is investigating the safety, tolerability, and efficacy of the treatment delivered by IA injection.

Canakinumab, a human anti-IL1β antibody, is currently in phase 2 of clinical evaluation, in combination with LNA043 (a protein inducing chondrogenesis and cartilage repair) for the treatment of patients with knee osteoarthritis (NCT04814368) after IA delivery.

#### 2.6.3. Conclusion and Perspectives Regarding the Intra-Articular Route

Intra-articular delivery may be of particular importance for the treatment of joint diseases (and notably rheumatoid arthritis) as around 30% of patients are resistant to biotherapies, and few novel molecules are under development. The limited half-life of antibodies reaching the inflamed joint is one of the major limitations associated with IA delivery, requiring frequent injections and thus increasing side effects, discomfort, and morbidity. Specific formulations including hydrogels [119], micro/nano particles [120], or in situ implants [121] are under consideration to improve antibody concentration on-target. A long-lasting Ab formulation through the use of biopolymers was recently developed for IA administration, showing a sustained high concentration of Abs in the synovial fluid, with minimal inflammatory side effects [122]. However, controlled-release methods are often associated with a loss of bioactivity of the drug, and/or inflammatory side effects that could accelerate the degeneration of the cartilage and the disease [123]. Additional work is needed to develop safe and active intra-articularly-delivered Abs to fight joints auto-immune diseases.

### 2.7. Delivery within the Central Nervous System: A Method to Bypass the Blood–Brain Barrier

#### 2.7.1. The Blood–Brain Barrier (BBB), a Barrier for Abs

The CNS is an essential part of the nervous system consisting of the brain and the spinal cord. The CNS integrates and coordinates essential functions of the body [124]. Because of its importance, the CNS is well protected, notably by the blood–brain barrier (BBB), limiting its exposure to exogenous particles carried by the blood circulatory system. The BBB is a highly selective semipermeable barrier preventing the passage of solutes from the blood into the extracellular fluid of the CNS. The BBB is composed of specialized endothelial cells sealed together with tight junctions reinforcing trans-endothelial electrical resistance, as well as the high expression of energy-dependent efflux transporter, inducing a selective passage of solutes [125]. In particular, FcRn, expressed in the microvascular endothelium and in the choroid plexus epithelium, essential components of the BBB, is involved in the reverse transcytosis of IgG, from the brain to the blood vessels. Therefore, it is estimated that less than 0.1% of systemically injected IgG enter the CNS through nonspecific pathways. Thus, to attain a therapeutic dose in the CNS, Abs have to be administered in high quantity—which may be associated with toxicity—or specific transporter pathways existing between the circulatory system and the CNS have to be used [126,127].

In order to circumvent these barriers, novel methods have been considered aimed at addressing the drug into the CNS by surgery, either via the intracerebroventricular, intracerebral routes or convection-enhanced delivery [126,128]. The intracerebral injection, the most direct method, consists in intermittent bolus injections, which are administered locally in the brain, after a surgical intervention. The intracerebroventricular technique allows the injection of drugs directly into the cerebrospinal fluid in the cerebral ventricles. Both methods use a syringe and needle system filled with the drug to be administered. Convection-enhanced delivery consists in the generation of a pressure gradient at the tip of an infusion catheter or canula (usually implanted in the cerebral tumor), allowing the delivery of drugs through the interstitial spaces of the CNS.

#### 2.7.2. Abs in Clinical Development for Direct Delivery to the CNS

There are many diseases targeting the CNS, including the neurodegenerative Parkinson or Alzheimer’s disease (AD), for which the development of Abs has been considered. For example, Aducanumab, an anti-Aβ Ab was approved by the FDA for the treatment of AD, after parenteral injection [129,130]. Cerebral delivery of Abs is mainly being evaluated in the preclinical phases with some success [131]; only one Ab has already reached clinical trials.

The antibody 131I-Omburtamab (Y-Abs Therapeutics), is a murine IgG1 recognizing CD276, used for radioimmunotherapy. This antibody is injected via the intracerebroventricular route, and phase 1 and 2 trials in patients with CNS Neuroblastoma, CNS metastases, or leptomeningeal metastases, have shown that the antibody was well tolerated and improved survival. A phase 3 trial is ongoing, evaluating the efficacy and safety of the Ab in children (NCT03275402). In the meantime, the same antibody is entering a phase 1 trial to test its efficacy, once delivered through a convention-enhanced delivery in patients with diffuse pontine gliomas profile (NCT05063357) [132].

#### 2.7.3. Conclusion and Perspectives on CNS Delivery

The CNS is particularly well protected from the environment impeding an efficient access to therapeutic molecules. The BBB remains the major obstacle, when considering systemic infusion of therapeutic Abs, and limits CNS bioavailability. Novel methods bypassing the BBB, described above, are still in infancy and require further work to be standardized and to reduce their invasiveness. In this context, the investigation of the “nose-to-brain” route may be of particular interest. It consists of administering drugs in the olfactory region of the nose, by inhalation, from where they may transfer to the brain through the epithelial layer and via neuronal bundles that project to the olfactory bulb. The “nose-to-brain” route is under preclinical evaluation for Abs [133].

## 3. Future Perspectives

If the pharmacokinetic and pharmacodynamic properties of therapeutic antibodies depend on their format and their target, they also depend on their route of administration. In fact, depending on the location of the Ab’s target, alternative modes of administration are being explored and developed to optimize Ab deposition in the vicinity of its target. The choice of route of administration is thus a critical factor for efficient Ab-based therapy and needs to be considered in the early stages of the development concomitantly with the appropriate formats, formulations, and devices (Table 9). If among the alternative routes some have progressed quite well to clinical trials, others are still at the very beginning of the research process. 

### 3.1. Oral Delivery: Protects Abs from the Harsh Environment of the Intestinal Tract

Some Abs have been approved over the years for the treatment of intestinal diseases, such as inflammatory bowel disease (IBD). However, their delivery through parenteral administration has displayed several limitations, such as non-responding patients, or the increase of anti-drug antibodies limiting the effect of the Abs. Oral delivery has been used for centuries and is the most accessible and the least invasive route for drug administration. Specific issues associated with the gastro-intestinal (GI) tract have to be considered to ensure efficient drug activity. The intestinal mucosa, in contact with external environment, maintains a state of homeostasis that sustains tolerance and detects and eliminates exogenous materials or pathogens. Within each part of the gastrointestinal tract, specific cellular and extracellular barriers exist, such as the harsh pH conditions, which denature Abs, and the presence of multiple proteases that may degrade proteins [134]. Studies have shown that less than 20% of Abs administered orally were still immunologically active after proteolytic digestion by gastric enzymes [135]. Thus, oral administration of Abs necessitates their protection them from the harsh conditions of intestinal transit.

Among the most advanced studies, AVX-470 is an orally delivered Ab targeting TNF, used to treat IBD. A phase 1 study concluded that the drug was safe and well-tolerated, and suggested a beneficial effect (NCT01759056) [136]. However, a high amount of AVX-470 was required because the majority of the antibody was degraded. This highlights the importance of protecting the antibody from the environment encountered in the GI tract.

One protection strategy may consist in encapsulating the Ab in either nanoparticles or enteric coated capsules, a strategy evaluated in a phase 1 trial for Foralumab—an anti-CD3 Ab—given to patients with active Crohn’s disease [137] (NCT05028946). Another strategy consists in decreasing the gastric acidic pH by combining the Ab with a proton pump inhibitor. An anti-CD3 (OKT3) was combined with omeprazole in patients suffering moderate to severe ulcerative colitis [138]. The six patients who received both OKT3 and omeprazole orally showed a promising reduction in inflammatory genes expression in peripheral blood cells, associated with low side effects.

Although studies have shown consistently promising results, it is important to better understand the fate of Abs once ingested. Strategies to bypass Abs degradation and uphold their activity remain to be investigated, and may also include the rational selection of the best isotypes which could survive to the extreme gastro-intestinal environment [139].

### 3.2. Skin Administration: A Topical Delivery of Ab in Wounds

Abs offer interesting opportunities to treat local skin diseases or wounds. While Abs may be administered directly to open wounds and allow direct healing, the treatment of blisters or scars would require their administration on intact skin [140]. However, when applied topically, antibodies showed low skin penetration, which may be explained by the hydrophobic nature of the keratin—essential and main component of the skin—and the hydrophilic nature of antibodies, and their size, limiting cellular and transcellular diffusion [141]. Thus, a high dose of antibody should be required to achieve a therapeutic dose inside the tissue. Moreover, for topical administration of drugs, gels or creams are recommended, which imply complex formulations of Abs to ensure their stability. Several strategies using cell-penetrating peptides, physical penetration enhancer, or injection with microneedles have been tested, but none of them has entered clinical trials.

## Figures and Tables

**Table 1:** Therapeutic antibodies approved by the Food and Drug Administration (FDA) or the European Medicine Agency (EMA) for subcutaneous administration. The routes of administration used to deliver an Ab affects its pharmacokinetics and pharmacodynamics, as well as exposing patients to potential harmful side effects. The systemic intravenous and subcutaneous routes are conventional routes for Abs, but novel local modes of administration are currently being investigated to improve the Ab therapeutic index. Optimal delivery of Abs may improve support for patients in numerous clinical situations. The figure was created with Biorender.com.

International Non-Proprietary Name	Trade Name	Target	Indication	Format	Sponsoring Company	Year of Approval (FDA)	Year of Approval (EMA)
Adalimumab	Humira	TNF-α	Rheumatoid Arthritis, Psoriatic Arthritis, and Ankylosing Spondylitis, Crohn disease	Human full-length IgG1	AbbVie Inc.	2002	2003
Efalizumab	Raptiva	CD11a	Psoriasis	Humanized full-length IgG1	Merck Biopharma/Serono Europe	2003 ^1^	2004 ^1^
Omalizumab	Xolair	IgE	Severe Asthma	Humanized full-length IgG1	Genentech/Novartis	2003	2005
Certolizumab Pegol	Cimzia	TNF-α	Crohn Disease	Humanized PEGylated Fab’ IgG1 fragment	UCB Pharma	2008	2009
Canakinumab	Ilaris	IL-1β	Cryopirin-Associated Periodic Syndromes	Human full-length IgG1	Novartis	2009	2009
Golimumab	Simponi	TNF-α	Rheumatoid Arthritis, Psoriatic Arthritis, and Ankylosing Spondylitis	Human full-length IgG1	Centocor Ortho Biotech/Janssen Biologics	2009	2009
Ustekinumab	Stelara	IL-12 and IL-23	Psoriasis	Human full-length IgG1	Centocor Ortho Biotech/Janssen Cilag International	2009	2009
Denosumab	Prolia	RANK-L	Postmenopausal Osteoporosis	Human full-length IgG2	Amgen	2010	2010
Tocilizumab	Actemra/RoActemra	IL-6R	Rheumatoid Arthritis, Polyarticular Juvenile Idiopathic Arthritis, Systemic Juvenile Idiopathic Arthritis	Humanized full-length IgG1	Genentech/Roche	2013	2014
Alirocumab	Praluent	PCSK9	LDL-C Lowering	Human full-length IgG1	Sanofi-Aventis	2015	2015
Evolocumab	Repatha	PCSK9	LDL-C Lowering	Human full-length IgG2	Amgen	2015	2015
Mepolizumab	Nucala	IL-5	Severe Asthma	Humanized full-length IgG1	GlaxoSmithKline	2015	2015
Secukinumab	Cosentyx	IL-17a	Psoriasis	Human full-length IgG1	Novartis	2015	2015
Daclizumab	Zinbryta	IL-2 receptor-α	Multiple Sclerosis	Humanized full-length IgG1	Biogen	2016 ^2^	2016 ^2^
Ixekizumab	Taltz	IL-17a	Psoriasis	Humanized full-length IgG4	Eli Lilly	2016	2016
Dupilumab	Dupixent	IL4 receptor-α	Atopic Dermatitis	Human full-length IgG4	Regeneron Pharmaceuticals/Sanofi-Aventis	2017	2017
Sarilumab	Kevzara	IL-6 receptor	Rheumatoid Arthritis	Human full-length IgG1	Regeneron Pharmaceuticals/Sanofi-Aventis	2017	2017
Guselkumab	Tremfya	IL-23 p19	Psoriasis	Human full-length IgG1	Janssen Biotech/Johnson & Johnson	2017	2017
Brodalumab	Siliq/Kyntheum	IL-17 receptor-α	Psoriasis	Human full-length IgG2	Valeant Pharmaceuticals/Leo Pharma	2017	2017
Benralizumab	Fasenra	IL-5 receptor-α	Severe Asthma	Humanized full-length IgG1	AstraZeneca	2017	2018
Emicizumab	Hemlibra	Factor IX and X	Hemophilia A	Humanized full-length bispecific IgG4	Genentech/Roche	2017	2018
Rituximab and hyaluronidase	Rituxan Hycela/MabThera SC	CD20	Follicular Lymphoma, Diffuse large B cell Lymphoma, and Chronic Lymphocytic Leukemia	Chimeric full-length IgG1	Genentech/Roche	2017	2014
Belimumab	Benlysta	BAFF	Systemic Lupus Erythematosus	Human full-length IgG1	GlaxoSmithKline	2017	2017
Tildrakizumab	Ilumya/Ilumetri	IL-23 p19	Psoriasis	Humanized full-length IgG1	Merck Sharp & Dohme Corp/Almirall	2018	2018
Burosumab	Crysvita	FGF23	X-linked Hypophosphatemia	Human full-length IgG1	Ultragenyx Pharmaceutical/Kyowa Kirin Holdings	2018	2018
Erenumab	Aimovig	CGRP	Migraine	Human full-length IgG2	Amgen/Novartis	2018	2018
Lanadelumab	Takhzyro	pKal	Hereditary Angioedema	Human full-length IgG1	Takeda Pharmaceuticals	2018	2018
Galcanezumab	Emgality	CGRP	Migraine	Humanized full-length IgG4	Eli Lilly	2019	2018
Caplacizumab ^3^	Cablivi	A1 from factor Willebrand	Acquired Thrombocytopenic Thrombotic Purpura	Humanized bivalent nanobody	Sanofi-Aventis	2019	2018
Romosozumab	Evenity	RANK-L	Postmenopausal Osteoporosis	Humanized full-length IgG2	Amgen/UCB Pharma	2019	2019
Trastuzumab and hyaluronidase	Herceptin Hylecta	HER2	HER2+ Breast Cancer	Humanized full-length IgG1	Genentech, inc./Roche	2019	2013
Risankizumab	Skyrizi	IL-23 p19	Psoriasis	Humanized full-length IgG1	AbbVie Inc.	2019	2019
Fremanezumab	Ajovy	CGRP	Migraine	Humanized full-length IgG2	Teva Branded Pharmaceutical Products R&D	2020	2019
Daratumumab and hyaluronidase	Darzalex Faspro	CD38	Light-chain Amyloidosis, Multiple Myeloma	Human full-length IgG1	Janssen Biotech	2020	2020
Satralizumab	Enspryng	IL-6 receptor	Neuromyelitis Optical Spectrum Disorder	Humanized full-length IgG2	Genentech/Roche	2020	2021
Ofatumumab	Kesimpta	CD20	Multiple Sclerosis	Human full-length IgG1	Novartis Pharmaceuticals Corporation	2020	2021
Pertuzumab, Trastuzumab, and hyaluronidase	Phesgo	HER2	Early Breast Cancer	Humanized full-length IgG1	Genentech/Roche	2020	2020
Tralokinumab	Adtralza	IL-13	Eczema	Human full-length IgG4	Leo Pharma A/S	2021	-
Tezepelumab	Tezspire	TSLP	Asthma	Human full-length IgG2	AstraZeneca	2021	-

^1^ The approval for Efalizumab was revoked, due to important side effects, by both the FDA and the EMA in 2009. ^2^ The approval for Daclizumab was revoked, due to important side effects, by both the FDA and the EMA in 2018. ^3^ The first dose of Caplacizumab is delivered through IV while the subsequent ones are delivered through SC. Data were obtained from the list of approved medicines and drugs by both agencies, and from the prescribing information provided by the manufacturers [28,29]. Abbreviations: TNF-α—Tumor Necrosis Factor alpha; IL—Interleukin; CD—Cluster of Differentiation; RANK-L—Receptor Activator of Nuclear factor Kappa-B ligand; PCSK9—Proprotein Convertase Subtilisin Kexin Type 9; BAFF—B-cell activating factor; FGF23—Fibroblast Growth Factor 23; Ig—Immunoglobulin; CGRP—Calcitonin gene-related peptide; PKal—Plasma Kallikrein; HER2—Human Epidermal Growth factor receptor 2; TSLP—Thymic Stromal Lymphopoietin.

**Table 2:** Therapeutic antibodies delivered by SC administration, currently undergoing review (URR) by the FDA and/or the EMA, and in active phase 3 of clinical development.

International Non- Proprietary Name or Code Name	Target	Indication	Format	Primary Sponsor	Clinical Study Phase	ClinicalTrials.gov—Identifiers
Casirivimab + Imdevimab	Spike protein	SARS-CoV2	Human full-length IgG1 cocktail	Regeneron Pharmaceuticals	URR (EMA + FDA)	NCT05074433
Ofatumumab ^1^	CD20	Relapsing multiple sclerosis	Human full-length IgG1	Novartis Pharmaceuticals	Phase 3 (Recruiting)	NCT04486716 NCT04510220 NCT04353492
CSL312 (Garadacimab)	Factor XIIa	Hereditary Angioedema	Human full-length IgG4	CSL Behring	Phase 3 (Active, not recruiting)	NCT04656418
SAR440340 /REGN3500 (Itepekimab)	IL-33	COPD	Human full-length IgG4	Sanofi	Phase 3 (Recruiting)	NCT04701983 NCT04751487
JS002 (Ongericimab)	PCSK9	Heterozygous Familial Hypercholesterolemia; Hyperlipidemia	Human full-length IgG4	Shanghai Junshi Bioscience Co., Ltd.	Phase 3 (Recruiting)	NCT05325203 NCT04781114
BCD-085 (Netakimab)	IL-17	Psioratic Arthritis	Humanized genetically modified IgG1	Biocad	Phase 3 (Active, not recruiting)	NCT03598751
AK002 (Lirentelimab)	Siglec-8	Eosinophilic Gastritis and/or Eosinophilic Duodenitis	Humanized non fucosylated IgG1	Allakos, Inc.	Phase 3 (Recruiting)	NCT05152563
MW032	RANK-L	Bone Metastases from solid tumors	Human full-length IgG2	Abwell (Shanghai) Bioscience Co., Ltd.	Phase 3 (Active, not recruiting)	NCT04812509
Tralokinumab ^1^	IL-13	Atopic Dermatitis	Human full-length IgG4	LEO Pharma	Phase 3 (Recruiting)	NCT05194540 NCT03587805
Golimumab ^1^	TNF-a	Moderately to Severely Active Ulcerative Colitis	Human full-length IgG1	Janssen Research & Development, LLC	Phase 3 (Recruiting)	NCT03596645
LY01011	RANK-L	Bone Metastases from solid tumors	Human full-length IgG2	Luye Pharma Group Ltd.	Phase 3 (Recruiting)	NCT04859569
QL1206	RANK-L	Bone Metastases from solid tumors	Human full-length IgG2	Qilu Pharmaceutical Co., Ltd.	Phase 3 (Recruiting)	NCT04550949
Adalimumab ^1^	TNF-a	Ulcerative Colitis	Human full-length IgG1	AbbVie	Phase 3 (Active, not recruiting)	NCT02632175
IBI306	PCSK9	Homozygous and Heterozygous Familial Hypercholesterolemia	Human full-length IgG2	Innovent Biologics (Suzhou) Co., Ltd.	Phase 3 (Recruiting)	NCT04031742 NCT04709536 NCT04179669
Alirocumab ^1^	PCSK9	Postprandial Hyperlipemia in Type 2 Diabetes	Human full-length IgG1	Regeneron Pharmaceuticals	Phase 3 (Recruiting)	NCT03344692
SYN023 (CTB-011 and CTB-012)	Proteins of human Rabies	Human Rabies	Humanized full-length IgG cocktail	Synermore Biologics (Suzhou) Co., Ltd.	Phase 3 (Active, not recruiting)	NCT04644484
Adalimumab ^1^	TNF-a	Uveitis	Human full-length IgG1	JHSPH Center for Clinical Trials	Phase 3 (Recruiting)	NCT03828019
CM310	IL-4Ra	Atopic Dermatitis	Humanized full-length IgG	Keymed Biosciences Co., Ltd.	Phase 3 (Not yet Recruiting)	NCT05265923
Benralizumab ^1^	IL-5Ra	Eosinophilic Gastritis and/or Gastroenteritis	Humanized full-length IgG1	AstraZeneca	Phase 3 (Recruiting)	NCT05251909
Elranatamab + Daratumumab	BCMA-CD3 + CD38	Multiple Myeloma	Humanized) bispecific IgG2 + Human full-length IgG1	Pfizer	Phase 3 (Recruiting)	NCT05020236
Teclistamab	BCMA-CD3+	Hematological Malignancies	Human bispecific IgG4	Janssen Research & Development, LLC	Phase 3 (Recruiting)	NCT03145181
PRO 140 (Leronlimab)	CCR5	HIV	Humanized full-length IgG4	CytoDyn, Inc.	Phase 3 (Active, not recruiting)	NCT03902522 NCT05271370 NCT02859961 NCT02990858
Olokizumab	IL-6	COVID-19	Humanized full-length IgG4	R-Pharm	Phase 3 (Recruiting)	NCT05187793
Etrolizumab	α4-β7/αE-β7 integrin receptor	Crohn’s disease	Humanized full-length IgG1	Hoffmann-La Roche	Phase 3 (Recruiting)	NCT02403323
Gantenerumab	β-amyloid	Alzheimer’s disease	Human full-length IgG1	Hoffmann-La Roche	Phase 3 (Recruiting)	NCT03444870 NCT05256134 NCT04374253 NCT01760005
Emicizumab ^1^	FIX, FX	Hemophilia A Without Inhibitor	Humanized bispecific IgG4	Margaret Ragni, University of Pittsburgh	Phase 3 (Recruiting)	NCT04303559

^1^ Those antibodies are already on the market, either delivered by another route, or for another application. Data were obtained from clinicaltrials.gov and EMA/FDA’s website. Abbreviations: IL—Interleukin; CD—Cluster of Differentiation; COPD—Chronic obstructive pulmonary disease; PSCK—Proprotein convertase subtilisin/kexin; RANK-L—receptor activator of nuclear factor kappa-B ligand; TNF—Tumor Necrosis factor; BCMA—B-cell maturation antigen; TSLP—Thymic stromal lymphopoietin; HNGF—Human nerve growth factor; CCR—C-C chemokine receptor; FIX—Factor IX; FX—Factor X.

**Table 3:** Therapeutic antibodies delivered by IM administration, currently undergoing regulatory review (URR) by the FDA and/or the EMA, or in active phase 3 of clinical development.

International Non- Proprietary Name or Code Name	Target	Indication	Format	Primary Sponsor	Clinical Study Phase	ClinicalTrials.gov—Identifiers
Tixagevimab/Cilgavimab (Mix of AZD8895 and AZD1061)	Spike protein of SARS-CoV2	COVID-19	Human full-length IgG1 cocktail	AstraZeneca	URR (EMA + FDA)	NCT04625725 NCT04625972 NCT04723394
Nirsevimab (MEDI8897)	Fusion (F) glycoprotein of RSV	Lower Respiratory Tract Infection (RSV)	Human full-length IgG1	AstraZeneca/MedImmune LLC	URR (EMA)	NCT02878330 NCT05110261
SYN023 (Mix of CTB011 and CTB012)	Residues found on RABV	Human Rabies	Humanized full-length IgG cocktail	Synermore Biologics (Suzhou) Co., Ltd.	Phase 3 (Active, not recruiting)	NCT04495569 NCT03961555 NCT04644484
MAD0004J08	SARS-CoV2	COVID-19	Human Fc-engineered IgG1	Toscana Life Sciences Sviluppo s.r.l.	Phase 3 (Active, not recruiting)	NCT04952805
VIR-7831 (Sotrovimab)	Spike protein of SARS-CoV2	COVID-19	Human full-length IgG1	Vir Biotechnology, Inc.	Phase 3 (Active, not recruiting)	NCT04913675
Ibalizumab-uiyk (Trograzo)	CD4-directed post-attachment inhibitor	HIV-1-infection	Humanized (from mouse) full-length IgG4	TaiMed Biologics Inc.	Phase 3 (Recruiting)	NCT03913195
MK-1654	Fusion Protein	RSV	Human full-length IgG1	Merck Sharp & Dohme Corp.	Phase 3 (Recruiting)	NCT04938830 NCT04767373
ADM03820	SARS-CoV2	COVID-19	Human Fc-engineered IgG1 cocktail	Ology Bioservices	Phase 2/Phase 3 (Not recruiting)	NCT05142527

Data were obtained from clinicaltrials.gov and EMA/FDA’s website. Abbreviations: RABV—Rabies virus; RSV—Respiratory Syncytial virus; H.I.V.—Human Immunodeficiency virus.

**Table 4:** Therapeutic antibodies approved by the Food and Drug Administration (FDA) and the European Medicine Agency (EMA) for intravitreal administration.

International Non- Proprietary Name	Trade Name	Target	Indication	Format	Sponsoring Company	Year of Approval (FDA)	Year of Approval (EMA)
Ranibizumab	Lucentis	VEGF-A	AMD, diabetic retinopathy, macular edema, branch, and central retinal vein occlusion	Humanized Fab IgG1 fragment	Genentech/Novartis Europharm	2006	2007
Brolucizumab	Beovu	VEGF-A	AMD	Humanized single-chain variable fragment IgG	Novartis	2019	2020
Faricimab	Vabysmo	VEGF-A and ANG-2	AMD, Diabetic Macular Edema	Humanized bispecific IgG1	Roche	2022	URR

Data were obtained from EMA/FDA’s website and from the prescribing information provided by the manufacturers [28,29]. Abbreviations: VEGF-A—Vascular Endothelial Growth Factor; AMD—Age-related Macular degeneration; ANG-2—Angiopoietin 2.

**Table 5:** Therapeutic antibodies in development, delivered by intravitreal/intraocular administration, currently in phase 3 clinical trial.

International Non- Proprietary Name or Code Name	Target	Indication	Format	Primary Sponsor	Clinical Study Phase	ClinicalTrials.gov—Identifiers
Faricimab	VEGF-A and ANG-2	nAMD, Macular edema secondary to BRVO	Humanized bispecific IgG1	Hoffmann-La Roche	Phase 3 (Recruiting)	NCT04777201 NCT04740905 NCT04740931 NCT04432831
Ranibizumab	VEGF-A	nAMD	Humanized IgG1 Fab fragment	Opthea Limited	Phase 3 (Recruiting)	NCT04757610
		CRVO With Macular Edema		University of Giessen	Phase 3 (Recruiting)	NCT04444492
		Retinopathy of Prematurity Both Eyes		Zagazig University	Phase 3 (Recruiting)	NCT05033106
		Diabetic Retinopathy, nAMD		Hoffmann-La Roche	Phase 3 (Active, not recruiting)	NCT04503551 NCT04657289 NCT05126966 NCT04108156 NCT03683251
		nAMD		King’s College Hospital NHS Trust	Phase 3 (Active, not recruiting)	NCT02243878
		Retinopathy of prematurity		Novartis Pharmaceuticals	Phase 3 (Active, not recruiting)	NCT02640664
Brolucizumab	VEGF-A	nAMD, Proliferative Diabetic Retinopathy	Humanized single-chain variable fragment IgG	Novartis	Phase 3 (Recruiting)	NCT04264819 NCT04239027 NCT04278417 NCT04058067 NCT04047472 NCT04005352
		Diabetic Macular Edema		Benha Univeristy	Phase 3 (Recruiting)	NCT04955171
Bevacizumab	VEGF-A	Diabetic Macular Edema	Humanized full-length IgG1	Laboratorios Sophia S.A de C.V.	Phase 3 (Recruiting)	NCT05217680
		Diabetic Macular Edema, Non-proliferative diabetic Retinopathy		Shahid Beheshti University of Medical Sciences	Phase 3 (Active, not recruiting)	NCT05083689 NCT04511715
		Retinopathy of Prematurity		Jaeb Center for Health Research	Phase 3 (Recruiting)	NCT04634604
		wAMD, BRVO, Diabetic Macular Edema		Outlook Therapeutics Inc.	Phase 3 (Active, not recruiting)	NCT05112861
		Retinal Telangiectasis, Coats Disease		Fondation Ophtalmologique Adolphe de Rothschild	Phase 3 (Recuiting)	NCT03940690
HLX04-O	VEGF-A	AMD	Humanized full-length IgG1	Shanghai Henlius Biotech	Phase 3 (Recruiting)	NCT05003245 NCT04740671
MW02	VEGF-A	wAMD	Humanized full-length IgG1	Mabwell (Shanghai) Bioscience Co., Ltd.	Phase 2 & 3 (Recruiting)	NCT05297292
KSI-301	VEGF-A	wAMDNon-Proliferative Diabetic RetinopathyRetinal Vein OcclusionDiabetic Macular Edema	Humanized biopolymer conjugate IgG1	Kodiak Sciences Inc	Phase 3 (Active, not recruiting)	NCT04049266 NCT05066230 NCT04592419 NCT04611152 NCT04603937 NCT04964089
LUBT010	VEGF-A	wAMD	Humanized IgG1 Fab fragment	Lupin Ltd.	Phase 3 (Recruiting)	NCT04690556

Data were obtained from clinicaltrials.gov and EMA/FDA’s website. Abbreviations: VEGF—Vascular Endothelial Growth factor; TNF—Tumor Necrosis Factor; AMD—Age-related Macular Degeneration; nAMD—Neovascular AMD; wAMD—Wet AMD; BVRO—Branch Retinal Vein Occlusion; CRVO—Central Retinal Vein Occlusion.

**Table 6:** Therapeutic antibodies in development delivered by intranasal/intratracheal administration, currently in phase 2 and phase 3 of clinical trial.

International Non- Proprietary Name or Code Name	Target	Indication	Format	Primary Sponsor	Clinical Study Phase	ClinicalTrials.gov—Identifiers
STI-2099 (COVI-DROPS)	SARS-CoV-2	COVID-19	Encoded plasmid DNA expressing IgG1 nAb	Sorrento Therapeutics, Inc.	Phase 2 (Active, not recruiting)	NCT05074394 NCT04906694 NCT04900428
CSJ-117 (Ecleralimab)	TSLP	AsthmaCOPD	Humanized IgG1 Fab Fragment	Novartis Pharmaceuticals	Phase 2 (Recruiting)	NCT04946318 NCT04882124
CT-P63 and CT-P59	SARS-CoV-2	COVID-19	Human IgG cocktail	Celltrion	Phase 3 (Recruiting)	NCT05224856
IBIO123	SARS-CoV-2	COVID-19	Human IgG cocktail	Immune Biosolutions Inc.	Phase 1 & 2 (Recruiting)	NCT05303376

Data were obtained from clinicaltrials.gov and the Embase online platform. Abbreviations: IL-4R—Interleukin receptor 4; TSLP—Thymic stromal lymphopoietin; nAb—Neutralizing Ab.

**Table 7:** Therapeutic antibodies in development delivered by intra-tumoral administration, currently in phases 2 and 3 of clinical trials.

International Non- Proprietary Name or Code Name	Target	Indication	Format	Primary Sponsor	Clinical Study Phase	ClinicalTrials.gov—Identifiers
APX005M (Sotigalimab)	CD40	Metastatic Melanoma	Humanized full-length IgG1	M.D. Anderson Cancer Center	Phase 1 & 2 (Recruiting)	NCT02706353
INCAGN01949	OX40	Locally Advanced Malignant Solid Neoplasm	Human full-length IgG1	University of Southern California	Phase 1 & 2 (Recruiting)	NCT04387071
Ipilimumab	CTLA-4	Stage III/IV melanoma	Human full-length IgG1	Gustave Roussy, Cancer Campus, Grand Paris	Phase 1 & 2 (Active, not recruiting)	NCT02857569
Checkpoint inhibitor such as Pembrolizumab	PD1/PDL1 and CTLA4	Solid Tumors	Humanized full-length IgG4	Second Affiliated Hospital of Guangzhou Medical University	Phase 2 & 3 (Recruiting)	NCT03755739
Urelumab	CD137	Neoplasms	Human full-length IgG4	Clinica Universidad de Navarra, Universidad de Navarra	Phase 1 & 2 (Recruiting)	NCT03792724

Data were obtained from clinicaltrials.gov and the Embase online platform. Abbreviations: CD40—Cluster of differentiation 40; OX40—Tumor necrosis factor receptor superfamily, member 4; CTLA-4—cytotoxic T-lymphocyte-associated protein 4; PD/PDL1—programmed cell death ligand and protein 1.

**Table 8:** Therapeutic antibodies in development delivered by intra-articular administration, currently in Phase 2 of clinical trials.

International Non- Proprietary Name or Code Name	Target	Indication	Format	Primary Sponsor	Clinical Study Phase	ClinicalTrials.gov—Identifiers
AMB-05X	c-FMS	Tenosynovial giant cell tumor, pigmented villonodular synovitis	Human full-length IgG2	AmMax Bio, Inc.	Phase 2 (Active, not recruiting)	NCT04731675 NCT05349643
Canakinumab	IL1 β	Knee osteroarthritis	Human full-length IgG1	Novartis Pharmaceuticals	Phase 2 (Recruiting)	NCT04814368

Data were obtained from clinicaltrials.gov and the Embase online platform. Abbreviations: c-FMS—colony stimulating factor I; IL—Interleukin.

**Table 9:** Advantages and limitations of the different routes of administration for Abs.

Routes of Administration	Advantages	Limitations
Subcutaneous	Allows self-delivery at home Allows longer delivery time and longer dosing intervals Less immunogenic	Poor prediction of bioavailability Needs specific formulation Slow drug absorption
Intramuscular	Abs can be absorbed through the circulatory systemic or though the lymphatic fluid Depot injection may allow longer release	Anatomy of muscle could lead to erratic absorption of Ab Invasive injection which may require assistance Limited to small volume
Intravitreal	Improves ocular bioavailability Devices allowing continuous delivery of Ab Good safety profile	Highly invasive Restricted list of excipients for formulation High inter-individual Abs pharmacokinetics
Inhalation	Rapid activity of Abs on-site, favorable for the treatment of respiratory diseases Low systemic absorption Easy to administer/self-administer	Multiple biological barriers High potential for aggregation of the Abs The real dose of drug that is bioavailable depends on the inhaler technique of patients
Intra-tumoral	Circumvention of extracellular barriers Limits on-target/off tumor effects	Dose and formulation need to be optimized Operator- and imaging-dependent
Intra-articular	Improves joint bioavailability Reduced systemic exposure	Limited half-life requiring frequent injections Long-term risks and benefits unknown
CNS delivery	Bypasses the BBB, limiting high and toxic concentration of systemic Abs “Nose-to-brain” method less invasive	Invasive procedure requiring surgery Limited diffusional capabilities

## Abbreviations

Ab, therapeutic antibody; ACE2, angiotensin-converting enzyme 2; AD, Alzheimer’s disease; AMD, age-related macular degeneration ANG-2, angiopoietin-2; BAFF, B-cell activating factor; BBB, blood–brain barrier; BCMA, B-cell maturation antigen; BVRO, Branch Retinal Vein Occlusion; CCR, C-C chemokine receptor; CD, cluster of Differentiation; c-FMS, colony-stimulating factor I; CGRP, calcitonin gene-related peptide; CNS, central nervous system; COPD, chronic obstructive pulmonary disease; CRVO, Central Retinal Vein Occlusion; CTLA-4, cytotoxic T-lymphocyte-associated protein 4; ECM, extra cellular matrix; EMA, European Medicines Agency; EUA, emergency use authorization; FDA, Food and Drug Administration; FGF23, Fibroblast Growth Factor 23; FVIII, Factor VIII; HER2, Human Epidermal Growth factor receptor 2; GI, gastro-intestinal; H.I.V., human Immunodeficiency virus; HNGF, human nerve growth factor; IA, intra-articular; IBD, inflammatory bowel disease; ICI, immune checkpoint inhibitor Ig, Immunoglobulin; IL, interleukin; IM, intra-muscular; IT, intra-tumoral IV, intravenous; mAb, monoclonal antibody; nAb, neutralizing Ab; nAMD, neovascular AMD; OA, osteoarthritis; OX40, Tumor necrosis factor receptor superfamily, member 4; PCSK9, Proprotein Convertase Subtilisin/Kexin Type 9; PDS, port delivery system; PD/PDL1, programmed cell death ligand and protein 1; pI, isoelectric point; PKal, Plasma Kallikrein; RABV, Rabies virus; RANK-L, receptor activator of nuclear factor Kappa-B ligand; RBD, receptor binding domain; RIG, (anti)-rabies immunoglobulin; RSV, Respiratory Syncytial Virus; SC, subcutaneous; TNF-α, tumor necrosis factor alpha; TSLP, thymic stromal lymphopoietin; URR, under regulatory review; VEGF, vascular endothelial growth factor; U.S., United States; wAMD, wet AMD.
